# Characterization of an intelectin-1 (*Itln1*) knockout mouse model

**DOI:** 10.3389/fimmu.2022.894649

**Published:** 2022-08-22

**Authors:** Eric B. Nonnecke, Patricia A. Castillo, Douglas T. Akahoshi, Stephanie M. Goley, Charles L. Bevins, Bo Lönnerdal

**Affiliations:** ^1^ Department of Microbiology and Immunology, School of Medicine, University of California, Davis, Davis, CA, United States; ^2^ Department of Nutrition, University of California, Davis, Davis, CA, United States

**Keywords:** omentin, lectin, IBD, innate immunity, mucosal immunity, tm1a, adipokine, Lieberkühn

## Abstract

Intelectins are carbohydrate-binding proteins implicated in innate immunity and highly conserved across chordate evolution, including both ascidians and humans. Human intelectin-1 (ITLN1) is highly abundant within the intestinal mucosa and binds microbial but not host glycans. Genome-wide association studies identified SNPs in *ITLN1* that are linked to susceptibility for Crohn’s disease. Moreover, ITLN1 has been implicated in the pathophysiology of obesity and associated metabolic disease. To gain insight on biological activities of human ITLN1 *in vivo*, we developed a C57BL/6 mouse model genetically targeting the gene encoding the functional mouse ortholog. In wild-type C57BL/6 mice, both mRNA and protein analysis showed high expression of *Itln1* in the small intestine, but manifold lower levels in colon and other extraintestinal tissues. Whereas intestinal expression of human ITLN1 localizes to goblet cells, our data confirm that mouse Itln1 is expressed in Paneth cells. Compared to wild-type littermate controls, mice homozygous for the *Itln1* hypomorphic trapping allele had reduced expression levels of *Itln1* expression (~10,000-fold). The knockout mice exhibited increased susceptibility in an acute model of experimentally induced colitis with 2% w/v dextran sulfate sodium (DSS). In a model of chronic colitis using a lower dose of DSS (1.5% w/v), which enabled a detailed view of disease activity across a protracted period, no differences were observed in body weight, fecal texture, hemoccult scores, food/water intake, or colon length at necropsy, but there was a statistically significant genotype over time effect for the combined fecal scores of disease activity. In model of diet-induced obesity, using two western-style diets, which varied in amounts of sugar (as sucrose) and saturated fat (as lard), mice with *Itln1* expression ablated showed no increased susceptibility, in terms of weight gain, food intake, plasma markers of obesity compared to wildtype littermates. While the mouse genetic knockout model for *Itln1* holds promise for elucidating physiological function(s) for mammalian intelectins, results reported here suggest that Itln1, a Paneth cell product in C57BL/6 mice, likely plays a minor role in the pathophysiology of chemically induced colitis or diet-induced obesity.

## Introduction

Intelectins (intestinal lectins) are a family of calcium-dependent secreted lectins implicated in innate immunity (1–6). Intelectins are highly conserved across chordate evolution and are abundantly expressed at mucosal surfaces, including the small intestine and colon of mammals (1, 7, 8). Human *ITLN1* encodes a lectin that binds acyclic vicinal (1,2)-diol moieties present on microbial but not mammalian glycans, suggesting that it may function in innate immunity as a pattern recognition binding protein (6, 9). *ITLN1*, a gene present on chromosome 1q23.3, has been identified by genome-wide association studies as a risk locus for Crohn’s disease (CD), a major form of inflammatory bowel disease (10–16). However, little is known about the *in vivo* function(s) of ITLN1 at the intestinal mucosa, or the mechanisms that may underlie its genetic association with CD (16). Moreover, human ITLN1, also known as “omentin,” is a highly abundant mRNA and protein product of visceral adipose and has been implicated in the pathophysiology of obesity and metabolically associated diseases as a “novel-adipokine” (17–20).

Mouse Itln1 appears to be a functional ortholog of human ITLN1, because like its human counterpart, mouse Itln1 selectively binds to acyclic vicinal (1,2)-diol moieties present on microbial glycans (9). Mouse Itln1 was initially discovered based on its high mRNA abundance in Paneth cells (8). Paneth cells are specialized secretory epithelial cells located in the small intestinal crypts of Lieberkühn that express an array of abundant and luminally secreted antimicrobial peptides and proteins, including α-defensins and lysozyme (21, 22). Whereas both mouse Itln1 and human ITLN1 are expressed in the small intestine, the human ortholog is not expressed in Paneth cells, but rather by a different secretory lineage - goblet cells (16, 20).

Like human ITLN1 and other mammalian homologs, little is known about the biological function of mouse Itln1. Mammalian intelectins exhibit variance in gene copy number, cellular localization, and oligomeric structure (1, 3, 16, 20, 23–25). Our strategy to further explore the effector functions of Itln1 was to generate a “knockout-first” targeting allele (tm1a) in C57BL/6 embryonic stem cells (26), which provides an allele designed for both hypomorphic expression of the targeted endogenous gene, as well as lacZ reporter gene expression driven by the target-gene promoter; subsequent site-specific genetic recombination in mice harboring the targeted allele can generate tissue-specific and whole-body gene deletions. We report here the development and analysis of mice harboring the *Itln1*-targeted trapping allele (designated *Itln1* ^trap^), where Itln1 expression is hypomorphic and the endogenous *Itln1* promoter drives LacZ expression. We further characterize the expression of *Itln1* and then examine outcomes of its ablation *in vivo* during experimentally induced colitis and diet-induced obesity.

## Materials and methods

### Mice

The Institutional Animal Care and Use Committee (IACUC) at the University of California, Davis, approved all procedures involving live animals and methods of euthanasia; experiments were performed in following AVMA guidelines and with adherence to IACUC-approved protocols. Mice were euthanized under deep anesthesia with ketamine (100 mg/kg) and xylazine (10 mg/kg).

### Generation of an intelectin-1 gene variant mouse

**Figure 1:**
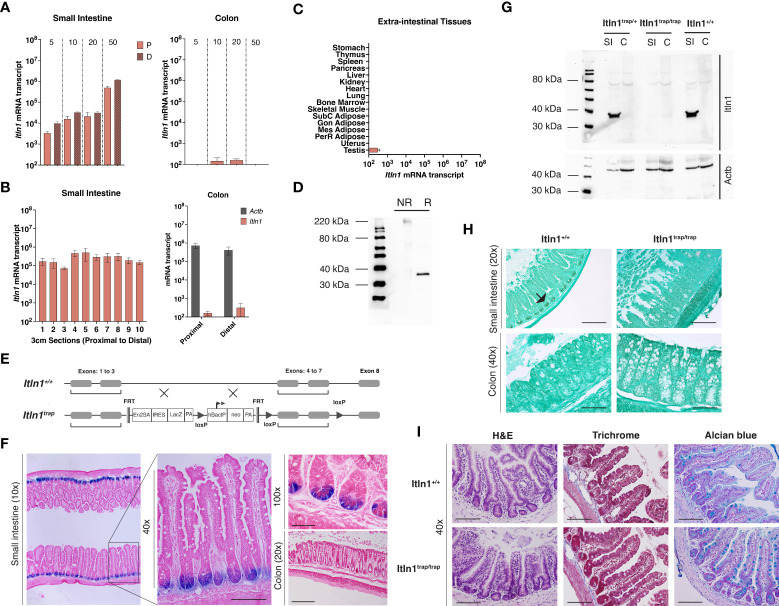
Characterization of intelectin-1 (*Itln1*) and an *Itln1* knockout model in C57BL/6N mice. **(A)** Quantitative RT-PCR analysis of *Itln1* mRNA in the proximal 10 cm (“P”) and distal 10 cm sections (“D”) of the small intestine (left) and whole colon (right) at post-natal day 5, 10, 20 and 50. **(B)** *Itln1* mRNA expression in 3 cm sections of small intestine arranged proximal (#1) to distal (#10) and colon divided into two equal length sections in adult mice. **(C)** Extra-intestinal tissue expression levels of *Itln1* mRNA. **(A-C)** Error bars represent standard error of the mean, n = 4 mice. **(D)** Immunoblot of Itln1 from small intestinal lysate: NR, non-reduced, denatured; R, reduced, denatured. **(E)** Schematic of *Itln1* ^trap^ cassette. **(F)** β-galactosidase staining in the small intestine of *Itln1* (*Itln1* ^trap/trap^) mice. The TM1A trapping cassette contains an internal ribosome entry site upstream of the reporter LacZ, which allows expression of β-galactosidase under control of the *Itln1* promoter. X-gal staining was detected in Paneth cells in the small intestinal crypts. β-galactosidase staining is absent in the colon of *Itln1* ^trap/trap^ mice. **(G)** A representative immunoblot of Itln1 in small intestine (SI) and colon **(C)** from *Itln1^+/+^*, *Itln1* ^trap/+^, and *Itln1* ^trap/trap^ mice. **(H)** Immunohistochemistry of Itln1 in Carnoy’s fixed small intestine and colon of *Itln1^+/+^* and *Itln1* ^trap/trap^ mice. Black arrows indicate small intestinal crypts. **(I)** Representative images of distal small intestine of *Itln1^+/+^* and *Itln1* ^trap/trap^ mice indicating absence of baseline inflammation (no cellular infiltrate or fibrosis; H&E and Masson’s trichrome) and similar appearance of crypts, villi, and cellular numbers (e.g., goblet cells; alcian blue) between genotypes. Scale bars: 20x = 200 µm, 40x = 100 µm, 100x = 50 µm.

The *Itln1* gene variant mouse strain was created in collaboration with the Mouse Biology Program at the University of California, Davis. Detailed methods used to create the tm1a allele have been reported previously (26), and the tm1a trapping-allele vector was generated by the trans-NIH Knock-Out Mouse Project (KOMP). Both the trapping allele vector and the C57BL/6N derived JM8.F6 embryonic stem (ES) cell line used for electroporation were obtained from the KOMP Repository (www.komp.org). The tm1a allele was designed to disrupt a critical region of the *Itln1* gene (**Figure 1E**). Within the tm1a (“trap”) allele, a human β-actin gene promoter is located upstream of a neomycin-resistance gene to enable clonal selection in the pool of ES cells. A second key design feature of the trapping allele is an internal ribosome entry site (IRES) upstream of LacZ reporter gene, which (i) interferes with expression of the native gene (insertion of the trapping cassette causes disruption of *Itln1* gene expression) and (ii) enables selective expression the LacZ reporter with specificity of the endogenous *Itln1* gene. For germline transmission, high percentage chimeric offspring (27, 28) were crossed with wild-type C57BL/6N mice to generate heterozygous *Itln1* ^trap/+^ colony founders on a pure C57BL/6N background.

### Genotype analysis

Tissue samples were digested in 25 mM NaOH/0.2 mM EDTA at 65°C overnight in a water bath, and then neutralized with 40 mM Tris HCl (pH 5.0). Isolated genomic DNA was amplified to detect the wild-type (WT, *Itln1^+^*) or the targeted *Itln1^trap^* allele using the following primers: *Itln1^trap^* (5’-GAGATGGCGCAACGCAATTAATG-3’, 5’-GAAAGCTAAAGCTAAACCCTGGGTGG-3’) and *Itln1^+^* (5’- AAGTCCTCTGATAGAGCAGTGCTTGC-3’, 5’- CAAGACCTGAAAGGCAGAAACAACC-3’). PCR: Briefly, sample DNA and primers were mixed with Taq DNA Polymerase, reaction buffer, and dNTP(s) according to the manufacturer’s protocol (New England Biolabs, Ipswich, MA). PCR reactions were as follows: 94°C for 5 min, 40 cycles [94°C 30 sec, 55°C 30 sec, and 72°C, 40 sec], and 72°C for 5 min. The PCR products were assessed by agarose gel electrophoresis, where the *Itln1^+^* allele produces a 340 BP product, and the *Itln1^trap^* allele produces a 439 BP product.

### RNA isolation, cDNA synthesis and quantitative real time PCR (qRT-PCR)

The general procedures for RNA isolation and synthesis of cDNA were previously described by our group (29). Briefly, tissue samples were dissected immediately upon euthanasia and placed in RNAlater (Ambion; Millipore Sigma, Burlington MA) incubated overnight at 4°C, and stored long-term at -20°C. Tissues were homogenized in guanidine thiocyanate buffer (29, 30). Total RNA was isolated using cesium chloride gradient ultracentrifugation (16, 29, 31), and then quantified using ultraviolet absorption spectrometry at 260 nm. For cDNA synthesis, 1 to 5 µg of total RNA was reverse transcribed using Superscript II reverse transcriptase (Invitrogen, Carlsbad, CA) using an oligo- (dT)12-18 primer (16, 29, 31). The single-stranded cDNA product was purified using Qiagen PCR purification kit (Qiagen, Valencia, CA), and diluted to 10 ng/µl based on the input concentration of total RNA. Primers specific for target genes (*Actb* [5’-GCTGAGAGGGAAATCGTGCGTG-3’, 5’-CCAGGGAGGAAGAGGATGCGG-3’], *Itln1* [5’-ACCGCACCTTCACTGGCTTC-3’, 5’- CCAACACTTTCCTTCTCCGTATTTC-3’], *Lyz1* [5’-GCCAAGGTCTACAATCGTTGTGAGTTG-3’, 5-CAGTCAGCCAGCTTGACACCACG-3’], and *Reg3g* [5’-CCTCAGGACATCTTGTGTC-3’, 5’- TCCACCTCTGTTGGGTTCA-3’) were designed using MacVector Software (MacVector, Cary, NC) (23, 31). Real-time quantitative PCR was performed as described previously (16, 29, 31) using a Roche Diagnostics Lightcycler 2.0 (Roche, Indianapolis, IN). The melting temperatures for PCR products of each reaction were determined to assure that they were indistinguishable from the cloned assay internal standard. Previous control experiments by our group demonstrated that when RNA from a single specimen was used to independently synthesize, isolate, and purify the cDNA, the reaction-to-reaction variability was ≤15% (29). Other reproducibility assessments of this approach were previously reported (29).

### Tissue *Itln1* mRNA expression

To characterize developmental expression patterns of *Itln1* in small intestine and colon, tissues were isolated from C57BL/6N mice at postnatal day (PD) 5, 10, 20, and 50 as previously described (31). Briefly, the small intestine was sectioned into proximal and distal portions. In adult mice (PD50, n = 4), 10 cm of proximal and distal small intestine was collected; full length of colonic tissue was also collected. In a separate experiment to investigate the topographical expression patterns of *Itln1* in adult mice, the small intestine (n = 4) was divided into 3 cm sections (n = 10 sections) proximally to distally (31). Extra-intestinal tissues of adult mice (n = 4) were similarly dissected for RNA extraction.

### Immunoblot analysis

General methods for western blot analysis have been reported (16, 20). Briefly, mouse intestinal tissue was homogenized in assay buffer (150 mM NaCl, 1% Triton X-100, 0.5% sodium deoxycholate, 0.1% SDS, 50 mM Tris, pH 8.0) containing a cocktail (1:100) of protease inhibitors (Protease Inhibitor Cocktail III; Calbiochem/EMD Millipore, Burlington, MA). Protein concentration was determined by bicinchoninic acid (BCA) assay (Pierce, ThermoFisher Scientific, Waltham, MA). Following heating at 95°C (either with or without reducing agent – 2-mercaptoethanol) for 30 min, protein (25 μg/lane) was loaded to a SDS-polyacrylamide gel (10% acrylamide) and electrophoresed for 40-50 min at 200 V, prior to wet transfer (Towbin Buffer: 25 mM Tris, 192 mM glycine, 20% v/v methanol, pH 8.3) to a nitrocellulose membrane (0.2 μM pore, Bio-Rad Laboratories, Hercules, CA) at 350 mA for 60-80 min. Estimated molecular size of Itln1 monomer was deduced using the MagicMark™ XP Western Protein Standard (ThermoFisher Scientific). The membrane was blocked in PBS-T (PBS with 0.1% Tween 20) containing 5% w/v skim milk for 30 min, and incubated with primary antibody at 4°C. Following overnight incubation, the membrane was washed with PBS-T for 1 hr, probed with donkey anti-rabbit IgG horseradish peroxidase (HRP) linked secondary antibody (GE Healthcare Amersham, Pittsburgh, PA) for 3 hr at room temperature, rinsed in PBS-T, and visualized using Femto and ECL West chemiluminescent substrates (ThermoFisher Scientific). Chemiluminescent signal was detected using a Biospectrum AC Imaging System (UVP, Upland, CA).

### Histology and immunohistochemistry

Mouse small intestinal and colonic tissues were fixed in 4% w/v paraformaldehyde (Sigma-Aldrich, St. Louis, MO), sequentially dehydrated in increasing concentration of ETOH and xylene, embedded in paraffin, and sectioned (4-5 μM) by microtome. Tissues were placed on X-tra™ positive-charged slides (Leica Biosystems, Buffalo Grove, IL). Slides were cleared of paraffin using xylene, washed with ETOH, and cleared of peroxidase activity by incubation in methanol containing 0.3% H_2_O_2_. Rehydrated samples were blocked with 5% v/v serum (donkey) in PBS prior to overnight incubation at 4°C with an in-house rabbit anti-ITLN antibody that recognizes a highly conserved region of mammalian intelectins, as previously reported (16, 20, 32, 33). Following incubation, slides were washed, and probed with a goat anti-rabbit secondary antibody and streptavidin-biotin ABC/horseradish peroxidase detection kit as outlined by the manufacturer (Vector Laboratories, Burlingame, CA). Slides were activated with 3,3- diaminobenzidine substrate (Vector Laboratories), and counterstained with light green (0.4% w/v in 0.2% glacial acetic acid) for 5 min prior to dehydration with denatured ETOH and xylene. Mounted slides were visualized using an Olympus BX51 microscope (Center Valley, PA). For histology, mounted tissue was stained with either hematoxylin and eosin, Masson’s trichrome or Alcian blue using standard protocols.

### LacZ tissue staining

Small intestinal and colonic tissues of 10-week-old Itln1^trap/trap^ homozygous mice were harvested, perfused with cold PBS, and fixed for 1 – 2 hr in fixation buffer (2% paraformaldehyde, 0.2% glutaraldehyde) at room temperature in a 6-well cell culture dish. LacZ staining was achieved using published protocols (34–36) and instructions outlined by the β-galactosidase Reporter Gene Staining Kit (Millipore-Sigma). Briefly, fixed tissue segments were washed with PBS and then incubated in the X-gal staining solution (5mM Potassium ferricyanide, 5mM Potassium ferrocyanide, 1mg/ml X-gal solution [X-gal dissolved in dimethylformamide]) for 14 – 24 hr at 37°C sealed and covered in foil. Following incubation (intestines appeared blue), the X-gal staining solution was removed, and tissues were washed three times in PBS (30 min each) under gentle rocking. Thereafter, tissues were further fixed with 4% paraformaldehyde overnight at 4°C, washed with PBS, and then transferred to tissue cassettes for paraffin embedding. Following tissue sectioning, specimens were cleared, rehydrated, and counterstained with eosin Y solution prior to visualization by light microscopy.

### Chemical colitis model

Study animals were provided *ad libitum* access to water and food (Purina Lab Diet 5001, Purina, St. Louis, MO). Heterozygous (*Itln1* ^trap/+^) breeding pairs on a pure C57BL/6N background were crossed to generate experimental wild-type (*Itln1* ^+/+^) and homozygous (*Itln1* ^trap/trap^) offspring. For the acute colitis model, singly housed male *Itln1* ^trap/trap^ and littermate *Itln1* ^+/+^ mice were administered 2% (w/v) dextran sulfate sodium (DSS: 36-50,000 Da; MP Biomedicals, Santa Ana, CA) as drinking water for 7 days, followed by pure H_2_O for an additional 6 days prior to necropsy. For the chronic colitis – carcinogenesis model, 12-week-old male *Itln1* ^trap/trap^ and *Itln1* ^+/+^ mice were injected intraperitoneally with 10 mg/kg body weight azoxymethane (Millipore-Sigma) one-week prior to the initial administration of 1.5% w/v DSS. The protocol for repeated DSS exposure was previously described with minor modifications (37, 38). Briefly, following the first DSS treatment for 7 d, animals were provided H_2_O for 14 d prior to the subsequent 7 d DSS treatment. In total, animals were challenged three times with DSS (7 d each) and provided H_2_O for an additional 14 d following the last challenge prior to necropsy. Animals were monitored for induction, progression, and resolution of disease across the study period. Body weight and fecal markers of disease activity were assessed daily. Feces were collected and scored for presence of occult blood (Hemoccult test kit, Beckman Coulter, Brea, CA) and consistency (texture and water content) based on an established scoring system (39). The scoring measures were as follows: stool texture, 0 (normal), 1 (increased moisture content), 2 (loose stool – soft but still discontinuous/formed), 3 (very soft/loose and/or continuous stool), and 4 (diarrhea, feces present on mouse anus/hindquarters); fecal blood: 0 (no blood), 1 (hemoccult positive but no visible blood), 2 (strong hemoccult positive and visible but discontinuous blood), 3 (visible, continuous blood), and 4 (gross bleeding and/or blood at anus). Intermediate scores of 0.5 were included where necessary. At necropsy, small intestinal length, colon length, and tumor count per colon were assessed.

### Diet-induced obesity model

8-week-old male wild-type (*Itln1* ^+/+^) and homozygous (*Itln1* ^trap/trap^) mice were randomized to receive one of three Research Diets (New Brunswick, NJ): control (CD; D12450K [10% kcal fat, 0% sucrose]), high fat-sugar (HFS; D12451 [45% kcal fat, 17% sucrose]), or very high fat (VHF; D12492 [60% kcals fat, 7% sucrose) for the duration of the experiment. Body weight and food intake were recorded daily. For the glucose tolerance test (GTT), male wild-type (*Itln1* ^+/+^) and homozygous (*Itln1* ^trap/trap^) mice fed CD (D12450K) for approximately 14 wk were fasted 6 hr (morning) prior to intraperitoneal injection with 2 g/kg body weight D-glucose (VetOne, Boise, ID) as described (40). Glucose concentrations were measured by glucometer (OneTouch Ultra2, LifeScan Inc., Milpitas CA) at t = 0-, 15-, 30-, 60-, and 120-min post glucose injection. For the insulin tolerance test (ITT), mice were fasted for 5 h prior to intraperitoneal injection with human recombinant insulin (0.75 U/kg body weight, Novolin, Princeton, NJ) (40). Blood collection procedures paralleled those of the GTT.

### Plasma adipokine analysis

At the experimental endpoint of the diet-induced obesity study, plasma was obtained from blood collected *via* cardiac venipuncture using EDTA-treated capillary tubes (Sarstedt, Newton, NC). Plasma analytes were assessed by Bio-Plex Multiplex Immunoassay (Bio-Rad, Hercules, CA). Sample preparation and assay procedure was performed as outlined by the manufactures’ guidelines.

### Statistical analysis

Statistical analysis was performed using Prism software, version 9.3.1 (GraphPad Software, Inc). Specific analysis details are indicated in figure legends.

## Results

### Itln1 expression and generation of a knockout mouse model

Using a quantitative real-time PCR assay, with an external plasmid standard of known concentration, the expression of intelectin-1 (*Itln1*) mRNA transcript in C57BL/6N mouse tissues was determined (29, 31). To characterize the expression patterns of *Itln1* in small intestine, we isolated samples from pre-weanling and adult mice. In both proximal and distal small intestine, *Itln1* mRNA expression across the pre-weaning period increased ~10-fold, and a further ~50-fold by postnatal day 50. Conversely, *Itln1* mRNA in the colon was present at manifold lower levels (~1,000 – 10,000-fold) and expression was independent of developmental period (**Figure 1A**). The patterns of *Itln1* mRNA expression in mouse intestine reported here mirrored other abundant Paneth cell products, which we previously described (31) such as *Lyz1 and* a subset of α-defensins (e.g., *Defa3*, *Defa23*, *and Defa26*). To delineate the proximal to distal expression of *Itln1* in the small intestine, we divided the small intestine into ten adjacent 3-cm sections and absolute quantity of *Itln1* mRNA was determined. Expression of *Itln1* was found to be similar (within threefold) across the length of the small intestine (**Figure 1B**). Additionally, at necropsy we dissected the colon of adult mice into two equal length portions (i.e., proximal and distal halves) to better characterize *Itln1* expression, as molecular regionalization has also been reported in murine colon (**Figure 1B**) (41). Constitutive *Itln1* mRNA expression was confirmed to be low in both regions. Using a threshold of detection for RNA transcripts of 10^2^ copies per 10 ng RNA, no expression of *Itln1* mRNA was detected in the extra-intestinal tissues tested, except testes (**Figure 1C**). Of note, two tissues with high *ITLN1* expression in humans, adipose and lung, were below the expression threshold for *Itln1* in mice (**Figure 1C**). These data are consistent with expression patterns of *Itln1* previously reported (23). Together, these results demonstrate that in C57BL/6 mice, *Itln1* mRNA expression is largely restricted to the small intestine.

Immunoblot analysis of small intestinal tissue extracts detected a band of ~35 kDa by SDS-PAGE under reducing conditions (**Figure 1D**), consistent with the expected size of a monomeric isoform based on the deduced amino acid sequence. Unlike human ITLN1, which forms disulfide-linked trimers, mouse Itln1 is predicted to generate only monomers, as it lacks the cysteine residues ascribed to trimer formation (24). Nevertheless, under non-reducing but denatured conditions, a single band was detected at ~220 kDa (**Figure 1D**). More work is required to further characterize this potentially higher-order oligomer that appears to migrate as a hexamer.

To investigate the biological activities of Itln1 *in vivo* we developed a gene variant mouse model on a pure C57BL/6 genetic background (see methods). A trapping allele (tm1a (26)) was designed to insert into and disrupt the *Itln1* gene (**Figure 1E**). The tm1a vector includes a neomycin resistance gene for clone selection, as well as FRT and LoxP sites (for future site-specific recombinase-mediated excision of the trapping cassette) and *Itln1* exons 4-7. The trapping allele has an internal ribosome entry site (IRES) upstream of the reporter LacZ (**Figure 1E**). Following germline transmission of the transgenic trapping allele (designated *Itln1* ^trap^), chimeric mice were bred to C57BL/6N mice, creating heterozygous mice - henceforth referred to as *Itln1* ^trap/+^. When crossed, *Itln1* ^trap/+^ mice generated offspring expected for Mendelian-inheritance patterns (data not shown; DNS). The gene expression of *Itln1* in mice homozygous for the trapping cassette (*Itln1* ^trap/trap^) had *Itln1* transcript levels reduced by ~10,000-fold in the small intestine compared to wild type littermate control mice (DNS).

Previous *in situ* hybridization analysis established that *Itln1* mRNA localizes specifically to Paneth cells in the small intestine of C57BL/6 mice (8). To confirm this cellular localization of Itln1 protein, we performed immunohistochemistry, and observed Itln1 staining in mouse intestinal crypts – indicative of Paneth cells (**Figure 1H**). By immunohistochemistry, Itln1 was undetectable in Paneth cells of *Itln1* ^trap/trap^ mice, and no Itln1 staining was observed in colons of either *Itln1* ^+/+^ or *Itln1* ^trap/trap^ animals in support of western blot and mRNA analysis (**Figure 1G**). The knockout *Itln1* ^trap/trap^ mice showed no signs of systemic or small intestine-specific pathology when unchallenged (**Figure 1I**), and the mice gained weight like *Itln1* ^+/+^ littermates (DNS and as observed in **Figure 3A**). The IRES of the trapping cassette enables expression of a LacZ reporter to be driven by the upstream endogenous *Itln1* gene promoter. Accordingly, expression of LacZ in *Itln1* ^trap/trap^ mice is evident in at the base of the small intestinal crypts (**Figure 1F**), consistent with established Paneth cell expression for *Itln1* mRNA by *in situ* hybridization (8).

### Chemical colitis

**Figure 2:**
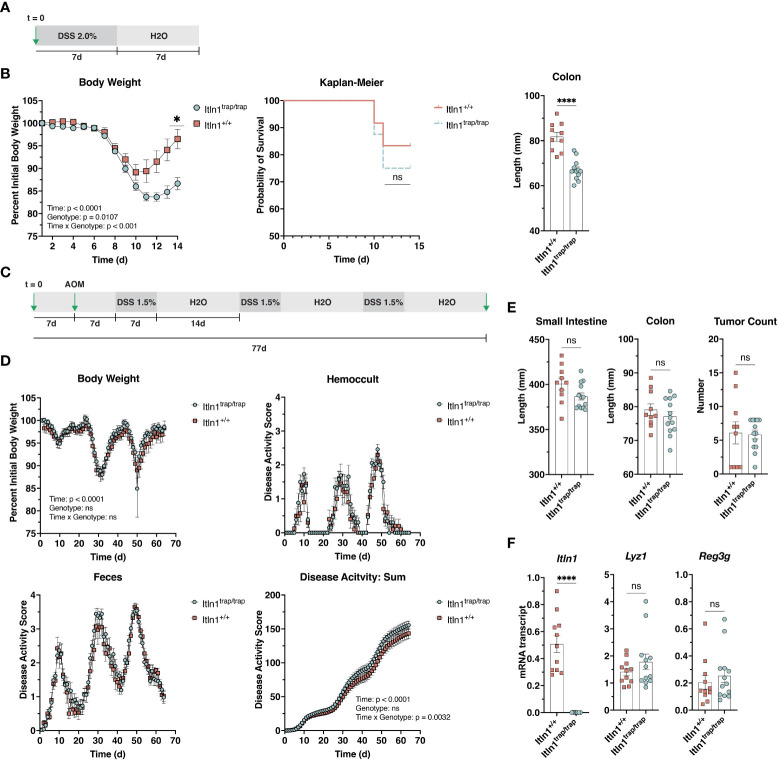
Response of Intelectin-1 knockout mice and wild-type littermates to challenge with DSS. **(A)** Treatment schematic for DSS colitis model. **(B)** Body weight, Kaplan-Meier survival curve, and colon length at necropsy (d 14) for *Itln1^+/+^* (n = 10-12) and *Itln1* ^trap/trap^ (n = 12-16) mice. **(C)** Treatment scheme for AOM-DSS chronic colitis model. **(D)** Body weight and disease activity parameters were assessed daily from the initial day of DSS administration to necropsy (total time = 63 d) in *Itln1^+/+^* (n = 10) and *Itln1* ^trap/trap^ (n = 13) mice. **(E)** Small intestine length, colon length, and colonic tumor number at necropsy. **(F)** Distal (last 5 cm) small intestine mRNA expression of Paneth cell effectors in *Itln1^+/+^* and *Itln1* ^trap/trap^ mice. Statistical analysis of body weight **(B)** and disease activity **(D)** was performed using repeated measures two-way ANOVA with Greisser-Greenhouse correction and Sidak multiple comparisons test. Survival analysis was performed by Chi square. Comparisons of tissue length and gene expression were performed using two-tailed, unpaired T-tests with Welch’s correction. p < 0.05 (*), p < 0.0001 (****), ns, non-significant.

Human ITLN1 is implicated in the etiology of inflammatory bowel disease (10–15). We assessed susceptibility to chemically induced colitis in *Itln1* ^trap/trap^ mice using a dextran sulfate sodium (DSS) model. *Itln1* ^trap/trap^ mice and *Itln1* ^+/+^ littermate controls were challenged with 2% w/v DSS in drinking water for 7 days. The DSS was then removed, and the mice were allowed to drink water until the termination of the experiment 6 days later (**Figure 2A**). Both knockout (*Itln1* ^trap/trap^) and wild type (*Itln1* ^+/+^) mice exhibited high sensitivity to 2% w/v DSS, including weight loss beginning at 7 d treatment (**Figure 2B**). Following DSS removal, animals continued to lose weight until day 10 at which point wild type mice began to regain weight. Compared to wild type control mice, *Itln1* ^trap/trap^ mice exhibited more pronounced weight loss, as well as impaired weight regain upon DSS removal and shortening of colonic length (**Figure 2B**). Mortality, which did not differ between genotypes, was approximately 15 – 25% (**Figure 2B**). Together, these results suggested that *Itln1* ^trap/trap^ mice are more susceptible to DSS.

Nevertheless, due to both the acute treatment period and relatively high mortality rate of mice receiving 2% DSS, we chose to pursue another established model of chronic colitis, which enabled a detailed view of disease activity across a protracted period (**Figure 2C**). Singly housed *Itln1* ^trap/trap^ mice and *Itln1* ^+/+^ littermate controls were pretreated with azoxymethane, and then challenged with three rounds of 1.5% w/v DSS (7 days). Between each DSS challenge, animals were provided DSS-free water for 14 d (**Figure 2C**). Body weight, disease activity (fecal consistency and hemoccult), and food/water intake were measured daily across the experimental period (63 days). Both *Itln1* ^+/+^ and *Itln1* ^trap/trap^ mice lost body weight and exhibited increase disease activity across the study period, where the second DSS treatment period resulted in a clear exacerbation (**Figure 2D**). At the lower DSS dose (1.5% w/v), no differences in body weight, the partitioned fecal texture and hemoccult scores, or food/water intake (DNS) were observed; however, there was a subtle but significant genotype over time effect for summation (combined fecal scores) of disease activity, suggesting *Itln1* ^trap/trap^ mice were slightly more sensitive to challenge (**Figure 2D**). At necropsy, neither small intestine nor colon lengths differed (**Figure 2E**). The colonic tumor burden induced by carcinogen azoxymethane appeared to be equivalent between the two groups (**Figure 2E**). Distal small intestine (last 5 cm) mRNA expression of Paneth cell effectors, *Lyz1* and *Reg3g* were similar between *Itln1* ^+/+^ and *Itln1* ^trap/trap^ mice (**Figure 2F**). Together, these results suggest that Itln1, a Paneth cell product in C57BL/6N mice, likely plays a minor role in the pathophysiology of chemically induced colitis.

### Diet-induced obesity

**Figure 3:**
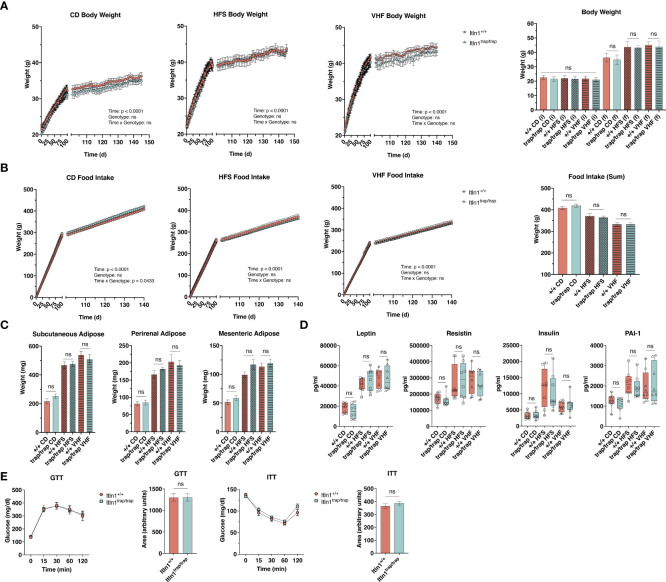
Response to obesity-inducing diets by intelectin-1 knockout mice. **(A)** Daily body weight and **(B)** food intake by *Itln1^+/+^* and *Itln1* ^trap/trap^ mice (n = 8 – 9/group). Animals were fed control (CD; 10% kcals fat, 0% sucrose), high-fat sugar (HFS; 45% kcals fat, 17% kcals sucrose), or very-high fat (VHF; 60% kcals fat, 7% kcals sucrose) chow for approximately 145 d. **(C)** Adipose depot weights and **(D)** serum adipokines at necropsy. **(E)** Intraperitoneal glucose- and insulin-tolerance tests, and associated area under the curve analyses in CD-fed animals (n = 6/genotype). Statistical analysis of daily body weight and food intake was performed using repeated measures two-way ANOVA with Greisser-Greenhouse correction and Sidak multiple comparisons test. Comparison of mean differences in body weight, food intake, adipose weight, and serum adipokines were performed using a one-way ANOVA with Tukey’s multiple comparisons test. Mean differences in glucose and insulin tolerance (area under the curve) were compared using two-tailed, unpaired T-tests. Displayed analyses represent genotype comparisons within diet groups. ns, non-significant.

Human ITLN1, expressed in visceral adipose, has been identified as a biomarker for obesity and related metabolic dysregulation (17–20, 42–45). To investigate the potential role of mouse Itln1 during diet-induced obesity, we assigned *Itln1* ^trap/trap^ mice and *Itln1* ^+/+^ littermates to receive either control or one of two western-style diets, with varied amounts of sugar (as sucrose) and saturated fat (as lard). Body weight and food intake was recorded daily for the duration of the experimental period (**Figures 3A, B**). *Itln1* ^trap/trap^ and *Itln1* ^+/+^ mice gained similar weight on each diet and consumed an equivalent amount of chow (**Figures 3A, B**). At necropsy, adipose depot mass was indistinguishable between genotype, reflective of body weight findings (**Figure 3C**). Furthermore, plasma concentrations of markers for obesity (leptin), inflammation (resistin), and insulin signaling (insulin and plasminogen activator inhibitor 1) did not differ between genotypes (**Figure 3D**). Lastly, in a cohort of control diet-fed animals, we investigated glucose and insulin tolerance to determine if under normal physiological conditions Itln1 plays a role in metabolic response. *Itln1* ^trap/trap^ and *Itln1* ^+/+^ mice demonstrated equivalent responses to glucose and insulin challenge (**Figure 3E**). Together, these findings demonstrate that ablation of Itln1 in C57BL/6 mice does not result in increased susceptibility to diet-induced obesity.

## Discussion

Mouse Itln1 is the apparent functional ortholog of human ITLN1 (9). In the present study we generated an *Itln1* knockout mouse model and tested the consequences in two *in vivo* models relevant to ITLN1 in human disease: inflammatory bowel disease (10–16) and obesity (17–20). C57BL/6 mice and related strains (e.g., C57BL/10), as well as respective sub strains (J and N) encode a single intelectin, *Itln1*. In contrast, other common laboratory strains (e.g., 129sv), as well as wild mice encode up to six *Itln1*-like genes (3, 23, 25). Our prior work demonstrated that *Itln1* is the primary intelectin gene constitutively expressed in the small intestine, regardless of mouse strain, and others identified Paneth cells as the expression site of *Itln1* by *in situ* hybridization (8, 23). Herein, using a mouse model developed on a C57BL/6N background, we confirmed that *Itln1* is expressed by Paneth cells of the small intestine. *Itln1* mRNA and protein expression was found largely restricted to this cell type, where *Itln1* mRNA transcript was essentially non-detectable at most extra-intestinal tissue sites, including lung, adipose, and colon (20). In contrast, the human ortholog ITLN1 is not expressed in Paneth cells, but rather is highly expressed in intestinal goblet cells (both small intestine and colon), as well as in visceral adipose tissue, among other sites (16). Intriguingly, the expression of human ITLN2, which has no clear structural/functional ortholog in murid rodents, is restricted to Paneth cells (20). Thus, intelectin expression in the small intestine differs between mice and humans, where mice express *Itln1* in Paneth cells, and humans express *ITLN1* in goblet cells and *ITLN2* in Paneth cells.

Mouse intelectin(s) 1-6 have been detected in other tissues (e.g., *Itln6* is constitutively expressed in colon), and some may be inducible (i.e., *Itln2* during parasitic infection) (3, 23, 25). Fortuitously, our knockout model was generated on a pure C57BL/6 background, which enabled complete intelectin ablation by targeting only a single gene, *Itln1*. However, an important caveat should be noted as C57BL/6 mice are an anomaly with respect to other laboratory and wild mice, which encode multiple intelectins. Therefore, extrapolation of findings related to *Itln1* should be considered in the context of mouse strain, where the additional intelectin paralogs may exert redundant or independent function(s) when present. Likewise, mouse Itln1, a functional ortholog of human ITLN1, is expressed by *i*) a different cell type in the small intestinal mucosa, and *ii*) not expressed at meaningful levels at extra-intestinal tissue sites.

ITLN1 has been identified in the context of multiple human diseases, including Crohn’s disease (CD) where nucleotide variants, associated with increased disease risk, have been identified at the *ITLN1* locus (10–16). The causal variant and associated outcome of this risk remains to be elucidated; however, altered expression of *ITLN1* within the intestinal mucosa does not appear to explain the associated risk haplotype (16). Furthermore, a missense variant (V109D), which is in linkage disequilibrium with GWAS-associated risk alleles, does not alter binding to an established microbial glycan binding partner, β-D-galactofuranose, or affect formation of higher-order oligomers (2, 9, 16). The latter two concepts (i.e., D109 and oligomerization) are delineating features between human ITLN1 and mouse Itln1, where D109 (i.e., amino acid tracking with the CD risk haplotype) is the ancestral amino acid found in mice and other mammalian ITLN1-orthologs. In terms of oligomerization, mouse Itln1 is predicted to be a monomer, due an absence of N-terminal cysteines that enable disulfide-linked homotrimer formation by human ITLN1 (24). Previous work by our group identified even higher oligomeric forms (i.e., hexamers) of not only human ITLN1, but also ITLN2, which may form through disulfide-independent mechanisms. In the present study, we demonstrated similar evidence for mouse Itln1 oligomerization, whereby in western blot analysis under non-reduced but denaturing conditions, a hexameric species was evident. The biological activities of lectins are classically dependent upon oligomeric form, and intelectins are not an exception, as human ITLN1 affinity to β-D-galactofuranose is enhanced in trimeric form (9, 46).

We investigated the response of *Itln1* knockout mice to challenge with DSS, a common model of chemically induced colitis in mice (38, 39). Using challenge with a high concentration of DSS (2% w/v), we observed increased susceptibility of the *Itln1* ^trap/trap^ knockout mice compared to *Itln1* ^+/+^ wild type littermates. This acute challenge resulted in relatively high mortality rate in both experimental groups. Therefore, we also tested a model of chronic colitis that uses a reduced DSS concentration (1.5% w/v), which allowed a prolonged assessment of disease activity. Although we identified a significant genotype across time effect, supporting the concept that *Itln1* ^trap/trap^ are more susceptible to DSS challenge, the overall effect was considered modest. DSS challenge is often viewed as a better experimental model of ulcerative colitis, rather than Crohn’s disease, with the later a heterogenous inflammatory disease of complex etiology occurring both in small intestine and colon (47–50). Human ITLN1 is implicated in innate immunity through its affinity for microbe-specific glycans (2, 6, 9). In theory, human ITLN1 and mouse Itln1 are present in the intestinal lumen, as both are produced by secretory epithelial cells; however, the noted differences between species in cellular and topographical expression may infer limitations on experimental models of human inflammatory bowel disease in mice that largely focus on the colon (39, 47). Luminal concentrations of intelectins in the small intestine and colon remain to be determined, as well as their specific activities, including the biological consequences if ITLN1-like proteins binding to targeted microbes and/or their carbohydrate constituents. Future work should aim to describe potential differences in the intestinal microbiota of *Itln1* knockout mice, to better understand the role of Paneth cell-derived *Itln1* in shaping host-microbe interactions.

In humans, in addition to expression by intestinal goblet cells, ITLN1 is also abundantly expressed in visceral adipose, where it is often referred to as “omentin.” In visceral adipose ITLN1 is expressed by an unknown cell type(s) of the stromal vascular fraction and is present in circulation at high nanomolar concentrations (17, 18, 42). ITLN1 has been proposed to have anti-inflammatory, and insulin-stimulating effects; however, specific mechanisms to explain such effects remain to be characterized (42, 45, 51). Serum ITLN1 concentrations have been evaluated in various disease states, including obesity, where an inverse relationship of ITLN1 concentration with adiposity has been observed (42, 43). In the present study, we investigated the effect of *Itln1* ablation on response to two obesogenic diets with varied levels of sugar and saturated fat. *Itln1* ^trap/trap^ knockout mice and *Itln1* ^+/+^ wild type littermates were found to be indistinguishable in every measure, including weight gain, food intake, and serum markers of adiposity and inflammation. Furthermore, *Itln1* ablation resulted in an unaltered response to glucose and insulin challenge under homeostatic conditions.

Alterations in Paneth cell biology, including endoplasmic reticulum stress and cytokine (e.g., type I interferon) signaling, have been observed in mice fed obesogenic diets – processes consistent with a diet-induced dysbiosis and role of Paneth cells in mediating host-microbe interactions through the production of various (highly abundant) effector molecules (21, 52–55). Our results suggest that Paneth cell derived Itln1 does not influence the onset and progression of diet-induced obesity in mice; however, additional work is required to better understand the potential role of Itln1 in mediating changes in the microbiota associated with high fat/sugar feeding. It is plausible that our model of dietary challenge (~20 wk) resulted in significant impairments of Paneth cell function in both knockout and wild type animals, thus mitigating the consequence of *Itln1* ablation. As outlined above, human ITLN1 and mouse Itln1 exhibit key differences in tissue expression. A key point from our analysis, supported by previous work, is that mice do not express *Itln1* (or other *Itln1*-like paralogs) in adipose at meaningful levels (i.e., >1000-fold lower than in small intestine), whereas human *ITLN1* mRNA transcript (i.e., expressed sequence tags) are more abundant than leptin, adiponectin, perilipin, and other adipose-associated genes in omental adipose (18, 20, 23). Taken together, the specific effector functions of human ITLN1 in visceral adipose cannot be easily addressed using a mouse model.

Human ITLN1 is implicated in the pathophysiology of both Crohn’s disease and metabolic disease, including obesity and associated type 2 diabetes (10–15, 17, 18, 42, 56). Major differences of mouse Itln1 expression in both tissue (e.g., visceral adipose) and cellular (e.g., Paneth cell vs. goblet cell) patterns compared to those for human ITLN1, unveiled here, provide precedent for additional studies focusing on small intestinal pathology (8, 16, 23). As opposed to chemical colitis, which targets the murine colon, genetic models of intestinal pathology, including the SAMP1/YitFc model of spontaneous ileitis might provide an attractive avenue to better study Itln1 in the context of Crohn’s disease; however, a confounding factor posed by this model is that it was developed on a mixed strain background derived from AKR/J inbred mice, which encode multiple *Itln* genes (i.e., *Itln1*, *-2*, *-4*, and *-6*) (23, 57–59). Alternatively, C57 congenic models, including SHIP^-/-^ (B6.129S6(C)-Inpp5dtm1.1Wgk/J) mice, which develop segmented, transmural ileitis due to perturbations in intestinal T cell immunity, present an opportunity to study Itln1 in isolation (60, 61). Furthermore, genetic models of neoplasia on a C57 background (e.g., C57BL/6J-*Apc^Min^*/J mice) may serve as better models than AOM-DSS to study the potential role of Itln1 in intestinal cancer (23, 62). In conclusion, while *Itln1* knockout mice on a C57BL/6 genetic background hold promise for interrogating physiological function(s) of this mammalian intelectin *in vivo*, the results reported here highlight several caveats that will need attention in the design and interpretation of experiments to model the physiology of the human protein.

## Data availability statement

The original contributions presented in the study are included in the article/supplementary material. Further inquiries can be directed to the corresponding authors.

## Ethics statement

The animal study was reviewed and approved by The Institutional Animal Care and Use Committee (IACUC) at the University of California, Davis.

## Author contributions

EN, CB, and BL designed research; EN, PC, DA, and SG performed research; all authors analyzed data; EN and CB wrote the paper; all authors edited the paper. All authors contributed to the article and approved the submitted version.

## Funding

National Institutes of Health U01AI125926 (Mucosal Immunology Study Team), R37AI32738, T32AI06055, U01HG004085, U01HG004080, and U42RR024244.

## Acknowledgments

We thank Xiaogu Du and James Graham for their valuable guidance on mouse colony development and modeling. We thank Sarah Bronstein, Sarah Winters, and Kevin Hoang for their assistance with data collection. We thank Professor Judith Van de Water and her laboratory members for assistance with the multiplex assay.

## Conflict of interest

The authors declare that the research was conducted in the absence of any commercial or financial relationships that could be construed as a potential conflict of interest.

## Publisher’s note

All claims expressed in this article are solely those of the authors and do not necessarily represent those of their affiliated organizations, or those of the publisher, the editors and the reviewers. Any product that may be evaluated in this article, or claim that may be made by its manufacturer, is not guaranteed or endorsed by the publisher.

## References

[1] Chen L Li J Yang G. A comparative review of intelectins. Scand J Immunol (2020) 92:e12882. doi: 10.1111/sji.12882 32243627

[2] Tsuji S Uehori J Matsumoto M Suzuki Y Matsuhisa A Toyoshima K. Human intelectin is a novel soluble lectin that recognizes galactofuranose in carbohydrate chains of bacterial cell wall. J Biol Chem (2001) 276:23456–63. doi: 10.1074/jbc.M103162200 11313366

[3] Pemberton AD Knight PA Gamble J Colledge WH Lee JK Pierce M. Innate BALB/c enteric epithelial responses to trichinella spiralis: inducible expression of a novel goblet cell lectin, intelectin-2, and its natural deletion in C57BL/10 mice. J Immunol (2004) 173:1894–901. doi: 10.4049/jimmunol.173.3.1894 15265922

[4] Voehringer D Stanley SA Cox JS Completo GC Lowary TL Locksley RM. Nippostrongylus brasiliensis: identification of intelectin-1 and -2 as Stat6-dependent genes expressed in lung and intestine during infection. Exp Parasitol (2007) 116:458–66. doi: 10.1016/j.exppara.2007.02.015 PMC2699772 17420014

[5] Kerr SC Carrington SD Oscarson S Gallagher ME Solon M Yuan S. Intelectin-1 is a prominent protein constituent of pathologic mucus associated with eosinophilic airway inflammation in asthma. Am J Respir Crit Care Med (2014) 189:1005–7. doi: 10.1164/rccm.201312-2220LE PMC4098098 24735037

[6] McMahon CM Isabella CR Windsor IW Kosma P Raines RT Kiessling LL. Stereoelectronic effects impact glycan recognition. J Am Chem Soc (2020) 142:2386–95. doi: 10.1021/jacs.9b11699 PMC7392083 31930911

[7] Wrackmeyer U Hansen GH Seya T Danielsen EM. Intelectin: a novel lipid raft-associated protein in the enterocyte brush border. Biochemistry (2006) 45:9188–97. doi: 10.1021/bi060570x 16866365

[8] Komiya T Tanigawa Y Hirohashi S. Cloning of the novel gene intelectin, which is expressed in intestinal paneth cells in mice. Biochem Biophys Res Commun (1998) 251:759–62. doi: 10.1006/bbrc.1998.9513 9790983

[9] Wesener DA Wangkanont K McBride R Song X Kraft MB Hodges HL. Recognition of microbial glycans by human intelectin-1. Nat Struct Mol Biol (2015) 22:603–10. doi: 10.1038/nsmb.3053 PMC4526365 26148048

[10] Barrett JC Hansoul S Nicolae DL Cho JH Duerr RH Rioux JD. Genome-wide association defines more than 30 distinct susceptibility loci for crohn's disease. Nat Genet (2008) 40:955–62. doi: 10.1038/ng.175 PMC2574810 18587394

[11] Franke A McGovern DP Barrett JC Wang K Radford-Smith GL Ahmad T. Genome-wide meta-analysis increases to 71 the number of confirmed crohn's disease susceptibility loci. Nat Genet (2010) 42:1118–25. doi: 10.1038/ng.717 PMC3299551 21102463

[12] de Lange KM Moutsianas L Lee JC Lamb CA Luo Y Kennedy NA. Genome-wide association study implicates immune activation of multiple integrin genes in inflammatory bowel disease. Nat Genet (2017) 49:256–61. doi: 10.1038/ng.3760 PMC5289481 28067908

[13] Jostins L Ripke S Weersma RK Duerr RH McGovern DP Hui KY. Host-microbe interactions have shaped the genetic architecture of inflammatory bowel disease. Nature (2012) 491:119–24. doi: 10.1038/nature11582 PMC3491803 23128233

[14] Ellinghaus D Jostins L Spain SL Cortes A Bethune J Han B. Analysis of five chronic inflammatory diseases identifies 27 new associations and highlights disease-specific patterns at shared loci. Nat Genet (2016) 48:510–8. doi: 10.1038/ng.3528 PMC4848113 26974007

[15] Liu JZ van Sommeren S Huang H Ng SC Alberts R Takahashi A. Association analyses identify 38 susceptibility loci for inflammatory bowel disease and highlight shared genetic risk across populations. Nat Genet (2015) 47:979–86. doi: 10.1038/ng.3359 PMC4881818 26192919

[16] Nonnecke EB Castillo PA Dugan AE Almalki F Underwood MA de la Motte CA. Human intelectin-1 (ITLN1) genetic variation and intestinal expression. Sci Rep (2021) 11:12889. doi: 10.1038/s41598-021-92198-9 34145348 PMC8213764

[17] Schäffler A Neumeier M Herfarth H Fürst A Schölmerich J Büchler C. Genomic structure of human omentin, a new adipocytokine expressed in omental adipose tissue. Biochim Biophys Acta (2005) 1732:96–102. doi: 10.1016/j.bbaexp.2005.11.005 16386808

[18] Yang RZ Lee MJ Hu H Pray J Wu HB Hansen BC. Identification of omentin as a novel depot-specific adipokine in human adipose tissue: possible role in modulating insulin action. Am J Physiol Endocrinol Metab (2006) 290:E1253–1261. doi: 10.1152/ajpendo.00572.2004 16531507

[19] Shibata R Ouchi N Takahashi R Terakura Y Ohashi K Ikeda N. Omentin as a novel biomarker of metabolic risk factors. Diabetol Metab Syndr (2012) 4:37. doi: 10.1186/1758-5996-4-37 22835063 PMC3411496

[20] Nonnecke EB Castillo PA Johansson MEV Hollox EJ Shen B Lönnerdal B. Human intelectin-2 (ITLN2) is selectively expressed by secretory paneth cells. FASEB J (2022) 36:e22200. doi: 10.1096/fj.202101870R 35182405 PMC9262044

[21] Bevins CL Salzman NH. Paneth cells, antimicrobial peptides and maintenance of intestinal homeostasis. Nat Rev Microbiol (2011) 9:356–68. doi: 10.1038/nrmicro2546 21423246

[22] Clevers HC Bevins CL. Paneth cells: maestros of the small intestinal crypts. Annu Rev Physiol (2013) 75:289–311. doi: 10.1146/annurev-physiol-030212-183744 23398152

[23] Almalki F Nonnecke EB Castillo PA Bevin-Holder A Ullrich KK Lönnerdal B. Extensive variation in the intelectin gene family in laboratory and wild mouse strains. Sci Rep (2021) 11:15548. doi: 10.1038/s41598-021-94679-3 34330944 PMC8324875

[24] Tsuji S Yamashita M Nishiyama A Shinohara T Li Z Myrvik QN. Differential structure and activity between human and mouse intelectin-1: human intelectin-1 is a disulfide-linked trimer, whereas mouse homologue is a monomer. Glycobiology (2007) 17:1045–51. doi: 10.1093/glycob/cwm075 17621593

[25] Lu ZH di Domenico A Wright SH Knight PA Whitelaw CB Pemberton AD. Strain-specific copy number variation in the intelectin locus on the 129 mouse chromosome 1. BMC Genomics (2011) 12:110. doi: 10.1186/1471-2164-12-110 21324158 PMC3048546

[26] Skarnes WC Rosen B West AP Koutsourakis M Bushell W Iyer V. A conditional knockout resource for the genome-wide study of mouse gene function. Nature (2011) 474:337–42. doi: 10.1038/nature10163 PMC3572410 21677750

[27] Pettitt SJ Liang Q Rairdan XY Moran JL Prosser HM Beier DR. Agouti C57BL/6N embryonic stem cells for mouse genetic resources. Nat Methods (2009) 6:493–5. doi: 10.1038/nmeth.1342 PMC3555078 19525957

[28] Coleman JL Brennan K Ngo T Balaji P Graham RM Smith NJ. Rapid knockout and reporter mouse line generation and breeding colony establishment using EUCOMM conditional-ready embryonic stem cells: A case study. Front Endocrinol (Lausanne) (2015) 6:105. doi: 10.3389/fendo.2015.00105 26175717 PMC4485191

[29] Wehkamp J Chu H Shen B Feathers RW Kays RJ Lee SK. Paneth cell antimicrobial peptides: topographical distribution and quantification in human gastrointestinal tissues. FEBS Lett (2006) 580:5344–50. doi: 10.1016/j.febslet.2006.08.083 16989824

[30] Chirgwin JM Przybyla AE MacDonald RJ Rutter WJ. Isolation of biologically active ribonucleic acid from sources enriched in ribonuclease. Biochemistry (1979) 18:5294–9. doi: 10.1021/bi00591a005 518835

[31] Castillo PA Nonnecke EB Ossorio DT Tran MTN Goley SM Lönnerdal B. An experimental approach to rigorously assess paneth cell α-defensin (Defa) mRNA expression in C57BL/6 mice. Sci Rep (2019) 9:13115. doi: 10.1038/s41598-019-49471-9 31511628 PMC6739474

[32] Suzuki YA Shin K Lonnerdal B. Molecular cloning and functional expression of a human intestinal lactoferrin receptor. Biochemistry (2001) 40:15771–9. doi: 10.1021/bi0155899 11747454

[33] Lopez V Kelleher SL Lonnerdal B. Lactoferrin receptor mediates apo- but not holo-lactoferrin internalization *via* clathrin-mediated endocytosis in trophoblasts. Biochem J (2008) 411:271–8. doi: 10.1042/BJ20070393 18171326

[34] Brown CB Feiner L Lu MM Li J Ma X Webber AL. PlexinA2 and semaphorin signaling during cardiac neural crest development. Development (2001) 128:3071–80. doi: 10.1242/dev.128.16.3071 11688557

[35] Barker N Clevers H. Lineage tracing in the intestinal epithelium. Curr Protoc Stem Cell Biol (2010) 13:5A.4.1-.11. doi: 10.1002/9780470151808.sc05a04s13 20443207

[36] Gierut JJ Jacks TE Haigis KM. Strategies to achieve conditional gene mutation in mice. Cold Spring Harb Protoc (2014) 2014:339–49. doi: 10.1101/pdb.top069807 PMC4142476 24692485

[37] Parang B Barrett CW Williams CS. AOM/DSS model of colitis-associated cancer. Methods Mol Biol (2016) 1422:297–307. doi: 10.1007/978-1-4939-3603-8_26 27246042 PMC5035391

[38] De Robertis M Massi E Poeta ML Carotti S Morini S Cecchetelli L. The AOM/DSS murine model for the study of colon carcinogenesis: From pathways to diagnosis and therapy studies. J Carcinog (2011) 10:9. doi: 10.4103/1477-3163.78279 21483655 PMC3072657

[39] Wirtz S Neufert C Weigmann B Neurath MF. Chemically induced mouse models of intestinal inflammation. Nat Protoc (2007) 2:541–6. doi: 10.1038/nprot.2007.41 17406617

[40] Ayala JE Samuel VT Morton GJ Obici S Croniger CM Shulman GI. Standard operating procedures for describing and performing metabolic tests of glucose homeostasis in mice. Dis Model Mech (2010) 3:525–34. doi: 10.1242/dmm.006239 PMC2938392 20713647

[41] Parigi SM Larsson L Das S Ramirez Flores RO Frede A Tripathi KP. The spatial transcriptomic landscape of the healing mouse intestine following damage. Nat Commun (2022) 13:828. doi: 10.1038/s41467-022-28497-0 35149721 PMC8837647

[42] de Souza Batista CM Yang RZ Lee MJ Glynn NM Yu DZ Pray J. Omentin plasma levels and gene expression are decreased in obesity. Diabetes (2007) 56:1655–61. doi: 10.2337/db06-1506 17329619

[43] Tan BK Pua S Syed F Lewandowski KC O'Hare JP Randeva HS. Decreased plasma omentin-1 levels in type 1 diabetes mellitus. Diabetes Med (2008) 25:1254–5. doi: 10.1111/j.1464-5491.2008.02568.x 19046210

[44] Moreno-Navarrete JM Ortega F Castro A Sabater M Ricart W Fernandez-Real JM. Circulating omentin as a novel biomarker of endothelial dysfunction. Obes (Silver Spring) (2011) 19:1552–9. doi: 10.1038/oby.2010.351 21293447

[45] Watanabe T Watanabe-Kominato K Takahashi Y Kojima M Watanabe R. Adipose tissue-derived omentin-1 function and regulation. Compr Physiol (2017) 7:765–81. doi: 10.1002/cphy.c160043 28640441

[46] Wesener DA Dugan A Kiessling LL. Recognition of microbial glycans by soluble human lectins. Curr Opin Struct Biol (2017) 44:168–78. doi: 10.1016/j.sbi.2017.04.002 PMC6688470 28482337

[47] Wirtz S Neurath MF. Mouse models of inflammatory bowel disease. Adv Drug Delivery Rev (2007) 59:1073–83. doi: 10.1016/j.addr.2007.07.003 17825455

[48] Valatas V Bamias G Kolios G. Experimental colitis models: Insights into the pathogenesis of inflammatory bowel disease and translational issues. Eur J Pharmacol (2015) 759:253–64. doi: 10.1016/j.ejphar.2015.03.017 25814256

[49] Rieder F Kessler S Sans M Fiocchi C. Animal models of intestinal fibrosis: new tools for the understanding of pathogenesis and therapy of human disease. Am J Physiol Gastrointest Liver Physiol (2012) 303:G786–801. doi: 10.1152/ajpgi.00059.2012 PMC4073977 22878121

[50] Baumgart DC Sandborn WJ. Crohn's disease. Lancet (2012) 380:1590–605. doi: 10.1016/S0140-6736(12)60026-9 22914295

[51] Greulich S Chen WJ Maxhera B Rijzewijk LJ van der Meer RW Jonker JT. Cardioprotective properties of omentin-1 in type 2 diabetes: evidence from clinical and *in vitro* studies. PloS One (2013) 8:e59697. doi: 10.1371/journal.pone.0059697 23555749 PMC3612072

[52] Guo X Tang R Yang S Lu Y Luo J Liu Z. Rutin and its combination with inulin attenuate gut dysbiosis, the inflammatory status and endoplasmic reticulum stress in paneth cells of obese mice induced by high-fat diet. Front Microbiol (2018) 9. doi: 10.3389/fmicb.2018.02651 PMC6230659 30455677

[53] Salzman NH Bevins CL. Dysbiosis–a consequence of paneth cell dysfunction. Semin Immunol (2013) 25:334–41. doi: 10.1016/j.smim.2013.09.006 24239045

[54] Liu TC Kern JT Jain U Sonnek NM Xiong S Simpson KF. Western Diet induces paneth cell defects through microbiome alterations and farnesoid X receptor and type I interferon activation. Cell Host Microbe (2021) 29:988–1001.e1006. doi: 10.1016/j.chom.2021.04.004 34010595 PMC8192497

[55] Guo X Li J Tang R Zhang G Zeng H Wood RJ. High fat diet alters gut microbiota and the expression of paneth cell-antimicrobial peptides preceding changes of circulating inflammatory cytokines. Mediators Inflammation (2017) 2017:9474896. doi: 10.1155/2017/9474896 PMC5339499 28316379

[56] Tan BK Adya R Randeva HS. Omentin: a novel link between inflammation, diabesity, and cardiovascular disease. Trends Cardiovasc Med (2010) 20:143–8. doi: 10.1016/j.tcm.2010.12.002 21742269

[57] Pizarro TT Pastorelli L Bamias G Garg RR Reuter BK Mercado JR. SAMP1/YitFc mouse strain: a spontaneous model of crohn's disease-like ileitis. Inflammation Bowel Dis (2011) 17:2566–84. doi: 10.1002/ibd.21638 PMC3154989 21557393

[58] Kosiewicz MM Nast CC Krishnan A Rivera-Nieves J Moskaluk CA Matsumoto S. Th1-type responses mediate spontaneous ileitis in a novel murine model of crohn's disease. J Clin Invest (2001) 107:695–702. doi: 10.1172/JCI10956 11254669 PMC208944

[59] Matsumoto S Okabe Y Setoyama H Takayama K Ohtsuka J Funahashi H. Inflammatory bowel disease-like enteritis and caecitis in a senescence accelerated mouse P1/Yit strain. Gut (1998) 43:71–8. doi: 10.1136/gut.43.1.71 PMC1727165 9771408

[60] Kerr WG Park MY Maubert M Engelman RW. SHIP deficiency causes crohn's disease-like ileitis. Gut (2011) 60:177–88. doi: 10.1136/gut.2009.202283 PMC3022365 20940287

[61] Wang JW Howson JM Ghansah T Desponts C Ninos JM May SL. Influence of SHIP on the NK repertoire and allogeneic bone marrow transplantation. Science (2002) 295:2094–7. doi: 10.1126/science.1068438 11896280

[62] Su LK Kinzler KW Vogelstein B Preisinger AC Moser AR Luongo C. Multiple intestinal neoplasia caused by a mutation in the murine homolog of the APC gene. Science (1992) 256:668–70. doi: 10.1126/science.1350108 1350108

